# The Potential and Concerns of Using AI in Scientific Research: ChatGPT Performance Evaluation

**DOI:** 10.2196/47049

**Published:** 2023-09-14

**Authors:** Zuheir N Khlaif, Allam Mousa, Muayad Kamal Hattab, Jamil Itmazi, Amjad A Hassan, Mageswaran Sanmugam, Abedalkarim Ayyoub

**Affiliations:** 1 Faculty of Humanities and Educational Sciences An-Najah National University Nablus Occupied Palestinian Territory; 2 Artificial Intelligence and Virtual Reality Research Center Department of Electrical and Computer Engineering An Najah National University Nablus Occupied Palestinian Territory; 3 Faculty of Law and Political Sciences An-Najah National University Nablus Occupied Palestinian Territory; 4 Department of Information Technology College of Engineering and Information Technology Palestine Ahliya University Bethlahem Occupied Palestinian Territory; 5 Centre for Instructional Technology and Multimedia Universiti Sains Malaysia Penang Malaysia

**Keywords:** artificial intelligence, AI, ChatGPT, scientific research, research ethics

## Abstract

**Background:**

Artificial intelligence (AI) has many applications in various aspects of our daily life, including health, criminal, education, civil, business, and liability law. One aspect of AI that has gained significant attention is natural language processing (NLP), which refers to the ability of computers to understand and generate human language.

**Objective:**

This study aims to examine the potential for, and concerns of, using AI in scientific research. For this purpose, high-impact research articles were generated by analyzing the quality of reports generated by ChatGPT and assessing the application’s impact on the research framework, data analysis, and the literature review. The study also explored concerns around ownership and the integrity of research when using AI-generated text.

**Methods:**

A total of 4 articles were generated using ChatGPT, and thereafter evaluated by 23 reviewers. The researchers developed an evaluation form to assess the quality of the articles generated. Additionally, 50 abstracts were generated using ChatGPT and their quality was evaluated. The data were subjected to ANOVA and thematic analysis to analyze the qualitative data provided by the reviewers.

**Results:**

When using detailed prompts and providing the context of the study, ChatGPT would generate high-quality research that could be published in high-impact journals. However, ChatGPT had a minor impact on developing the research framework and data analysis. The primary area needing improvement was the development of the literature review. Moreover, reviewers expressed concerns around ownership and the integrity of the research when using AI-generated text. Nonetheless, ChatGPT has a strong potential to increase human productivity in research and can be used in academic writing.

**Conclusions:**

AI-generated text has the potential to improve the quality of high-impact research articles. The findings of this study suggest that decision makers and researchers should focus more on the methodology part of the research, which includes research design, developing research tools, and analyzing data in depth, to draw strong theoretical and practical implications, thereby establishing a revolution in scientific research in the era of AI. The practical implications of this study can be used in different fields such as medical education to deliver materials to develop the basic competencies for both medicine students and faculty members.

## Introduction

### Background

Artificial intelligence (AI) has many applications in various aspects of our daily life, including health, criminal, education, civil, business, and liability law [1,2]. One aspect of AI that has gained significant attention is natural language processing; this refers to the ability of computers to understand and generate human language [3]. As a result, AI has the potential to revolutionize academic research in different aspects of research development by enabling the analysis and interpretation of vast amounts of data, creating simulations and scenarios, clearly delivering findings, assisting in academic writing, and undertaking peer review during the publication stage [4,5].ChatGPT [6], one of the applications of AI, is a variant of the GPT language model developed by OpenAI and is a tool designed to generate humanlike text in a conversational style that can engage in conversations on various topics. Trained on human-human conversation data, ChatGPT can generate appropriate responses to questions and complete discussions on its own, making it a valuable tool for natural language processing research. As a language model developed by OpenAI, ChatGPT has been widely used in various fields, such as language translation, chatbots, and natural language processing [7]. ChatGPT has many applications in multiple domains, including psychology, sociology, and education; additionally, it helps to automate some of the manual and time-consuming processes involved in research [8]. Furthermore, its language-generation capabilities make it a valuable tool for natural language processing tasks, such as summarizing complex scientific concepts and generating scientific reports [9]. The features of ChatGPT make it an attractive tool for researchers whose aim is to streamline their workflow, increase efficiency, and achieve more accurate results.

### Research Gap

Many studies, preprints, blogs, and YouTube (YouTube, LLC/Google LLC) videos have reported multiple benefits of using ChatGPT in higher education, academic writing, technical writing, and medical reports [10]. However, many blogs have raised concerns about using ChatGPT in academic writing and research. Moreover, some articles consider ChatGPT to be an author and have listed it as a coauthor; this has raised many questions regarding research integrity, authorship, and the identity of the owners of a particular article [11]. What has been written regarding the issue of using ChatGPT as a research generator has been limited to opinions and discussions among researchers, editors, and reviewers. Various publishers have organized these discussions to explore possible agreements and the development of ethical policies regarding the use of ChatGPT in academic writing and research. However, there are limited practical studies on using ChatGPT in scientific research. There is a gap in understanding the potential, limitations, and concerns of using ChatGPT in scientific research as well as the ethical and social implications of incorporating AI in scientific work.

Additionally, there is a lack of standardized methods and best practices for using ChatGPT in scientific research. These gaps highlight the need for further research to investigate the effectiveness, accuracy, and trustworthiness of ChatGPT when used for scientific research, as well as to identify the ethical and societal implications of using AI in scientific inquiry. It is important to investigate the concerns of using ChatGPT in academic research to ensure safe practices and consider the required ethics of scientific research.

### Purpose of the Study

This paper aims to examine the role of ChatGPT in enhancing academic performance in scientific research in social sciences and educational technology research. It also seeks to provide insights and guidance for researchers in different fields such as in medical sciences, specifically research in medical education.

### Contribution of the Study

Conducting scientific research regarding the potential for ChatGPT to be used in scientific research could provide researchers with valuable insights into the capabilities, benefits, and limitations of using AI in research. The findings of this research are expected to help identify the potential bias and ethical considerations associated with using AI to inform future developments and assess the implications of using AI in scientific research on different topics and multidisciplinary research fields such as medical education. Moreover, by exploring the use of ChatGPT in specific fields, such as social sciences and educational technology, researchers can gain a deeper understanding of how AI can enhance academic performance and support advancing knowledge in various education fields such as engineering and medical education. In the context of this study, the researchers consider ChatGPT to be an e-research assistant, or a helpful tool, for researchers to accelerate the productivity of research in their specific areas. Therefore, this study attempts to answer the following research questions:

- What are the best practices for using AI-generated text, specifically ChatGPT, in scientific research?
- What are the concerns about using AI-generated text, specifically ChatGPT, in academic research?

### AI-Generated Text Revolutionizing Medical Education and Research

AI-generated text, powered by AI technologies such as ChatGPT, has revolutionized medical education and research. It offers unique opportunities to enhance learning experiences and provide medical professionals with up-to-date knowledge [12]. Its integration into medical education brings several advantages, such as real-time access to vast medical information, continuous learning, and evidence-based decision-making [13]. AI-generated text bridges the knowledge gap by providing accurate and current information from reputable sources, facilitating access to relevant medical literature, research studies, and clinical guidelines [14]. This personalized learning tool fosters critical thinking and self-directed exploration of medical concepts, while also offering instant feedback and adaptive learning experiences [15].

In medical research, ChatGPT plays a crucial role by assisting researchers in gathering and analyzing extensive medical literature, saving valuable time and effort [12,13]. It fosters collaboration among researchers and aids in data analysis, uncovering patterns and relationships within data sets [14]. Moreover, ChatGPT facilitates the dissemination of research findings by creating accessible summaries and explanations of complex work [15]. This promotes effective communication between research and clinical practice, ensuring evidence-based health care practices.

Despite its benefits, AI-generated text presents challenges in medical education and research. Ensuring the reliability and accuracy of information is essential, considering the potential for incorrect or misleading data [12]. Validating sources and aligning content with medical standards and guidelines are crucial steps. Additionally, AI-generated text may lack human interaction, which is vital for developing communication and empathy skills in medical practice [13]. To strike a balance, medical education should combine AI-generated text with traditional teaching methods, emphasizing direct patient interaction and mentorship [14].

Researchers must exercise caution when relying on AI-generated text, critically evaluating the information provided [15]. Human expertise and judgment remain indispensable to ensure the validity and ethical considerations of research findings [16]. Thoughtful integration of AI-generated text in medical education and research is essential to harness its potential while preserving the essential human touch required in medical practice [17].

In conclusion, AI-generated text has transformed medical education and research by offering accessible and up-to-date knowledge to medical professionals [12]. It empowers learners to engage in self-directed exploration and critical thinking, while also providing personalized feedback for improvement [15]. In research, ChatGPT aids in data analysis, communication, and dissemination of findings, bridging the gap between research and practice [15]. However, careful consideration of its reliability and integration with human expertise is crucial in both medical education and research settings [12]. By embracing AI-generated text thoughtfully, the medical field can leverage its potential to drive innovation and advance evidence-based health care practices [16].

### Exploring the Application of ChatGPT in Scientific Research

Researchers can use ChatGPT in various ways to advance research in many fields. One of the applications of ChatGPT in scientific research is to provide researchers with instructions about how to conduct research and scientific research ethics [18].

Researchers can ask the application to provide a literature review in a sequenced way [19]. ChatGPT can organize information into tables based on the prompts used by researchers and the flow of the research stages [20]. Moreover, researchers can utilize ChatGPT’s ability to summarize data and write reports based on detailed data; the application also makes it easier for researchers and analysts to understand and communicate their findings. Practitioners and researchers have already been using language models such as ChatGPT to write, summarize published articles, talk, improve manuscripts, identify research gaps, and write suggested research questions [4]. Moreover, practitioners use AI-generated text tools to generate examination questions in various fields, while students use them to write computer code in competitions [18]. AI will soon be able to design experiments, write complete articles, conduct peer reviews, and support editorial offices accepting or rejecting manuscripts [21]. However, researchers have raised concerns about using ChatGPT in research because it could compromise the research integrity and cause significant consequences for the research community and individual researchers [22].

Although the development of ChatGPT has raised concerns, it has provided researchers with opportunities to write and publish research in various fields. Researchers can learn how to begin academic research; this is especially relevant to novice researchers and graduate students in higher education institutions. Various reports have been released regarding using ChatGPT when writing student essays, assignments, and medical information for patients [7]. However, many tools have also been released to look for and identify writing undertaken by ChatGPT [23,24].

ChatGPT has limitations when writing different stages of academic research; for example, the limitation of integrating real data in the generated writing, its tendency to fabricate full citations, and the fabrication of knowledge and information relating to the topic under investigation [7].

### Understanding the Legal Landscape of ChatGPT

It was stated above that ChatGPT is an AI language model developed and owned by OpenAI, which can be used in higher education, academic writing, and research [10,18]. However, it is noticeable that the ChatGPT model does not currently include any terms and conditions, or a fair use policy per se, published on its system or website. Meanwhile, it is important to note that this situation may change in the future, as the model’s owner may, at any time, apply or enforce their policies, terms, and conditions, or membership and charges as they see fit. Nevertheless, the absence of any regulatory terms or policies on using ChatGPT should not subsequently mean that there are no other legal or ethical regulations that researchers must consider and follow when using ChatGPT services. Rules concerning data protection and intellectual property rights are equally relevant to protecting both the rights of the owner of the ChatGPT system and the intellectual property rights of authors that ChatGPT has sought and from whose work it has generated its information [25].

As to the rules concerning the property rights of the owner of the ChatGPT system, it is a criminal offense for anyone to engage in any harmful activity, misuse, damage, or cyberattack on the system and its operation [26,27]. Cybercrimes and copyright infringements may refer to any activity that is considered illegal under domestic or international criminal law [28,29].

For example, using malicious tactics to cause damage to ChatGPT systems and user-mode applications; engaging in data theft, file removal or deletion, and digital surveillance; or attempting to gain external remote control over the ChatGPT system can be considered illegal acts that may result in criminal charges under national or international law [28,30].

Furthermore, it should be noted that cybercrimes and copyright infringements carry potential criminal consequences and civil liabilities [31]. This means that the owner of the ChatGPT system and any other third party affected by such acts have the right to seek remedies, including compensation, under the Law of Tort [32]. These rights are implied under national and international law and do not need to be explicitly stated on the ChatGPT website or within its system [33,34].

As to the second point relating to respecting intellectual property rights, while ChatGPT and its owners are generally not responsible for how a person uses the information provided by the system, it is the user’s liability if any information obtained from the system in any way constitutes a breach of national law, or could lead to a criminal conviction. This could include, for example, the commission of fraud, cyberbullying, harassment, or any other activity that violates an individual country’s applicable laws or regulations [27,30]. Suppose a person or group of people have published misleading or deceptive information. In that case, they may be at risk of being charged with a criminal offense [35], even though such information is gathered from the ChatGPT model. Accordingly, it remains the sole responsibility of every individual using the operation of ChatGPT to ensure that the information gathered or provided by the system is accurate, complete, and reliable. This means that it remains the sole responsibility of every individual to ensure that any information published or provided to others is done so in accordance with the applicable national or international law, including that related to intellectual property rights, data protection, and privacy of information [35,36].

Concerning academic writing and research, it is also important to note that researchers are responsible for and expected to follow ethical and professional standards when conducting, reporting, producing academic writing, or publishing their research [25,37]. Accordingly, if a researcher relies on ChatGPT in whole or in part for scientific research, the attribution of the information gathered would depend on the specific context and circumstances of the research. For example, research cannot provide scientific information discovered by others without appropriate reference to the original research [38]. The researcher cannot claim intellectual property rights or provide misleading information that may infringe on the proprietary rights of others [36,39].

Intellectual property rights are governed by national and international law and practice. Researchers using ChatGPT as a source in their manuscript writing are familiar with all ethical and legal policies and internal and international laws regulating their work, including copyright protection, privacy, confidentiality protection, and personal property protection. Therefore, users of ChatGPT should not rely on the service to engage in any activity that infringes upon the intellectual property rights of others, including but not limited to copyright, trademark, or privacy infringement [30]. Nevertheless, where data or published research is concerned, protecting intellectual property rights is not limited to one individual copyright but usually involves protecting the rights and interests of all members connected to the data and published research [40]. This includes but is not limited to educational institutions, government institutions or authorities, private sectors, and funding institutions [41]. This means that all parties involved in data collection, research publication, and other published information from which ChatGPT has gathered its information must be cited, attributed, and acknowledged in the manuscript submitted for publication. This is an ethical requirement and involves copyright and intellectual property rights [39].

In short, when using ChatGPT, researchers must be mindful of the legal and regulatory requirements related to their use of the service, including those relating to intellectual property rights, data protection, and privacy [42]. When using ChatGPT for research purposes, researchers should pay serious attention to the potential ethical implications of their research and take steps to ensure that their use of the service is responsible and in compliance with relevant standards and guidelines [43]. Ultimately, the responsibility for conducting research lies with researchers; they should follow best practices and scientific ethics guidelines in all research phases.

Based on a conversation between researchers participating in the study and ChatGPT, the application claims that it has revolutionized academic research writing and publishing within a short time compared with traditional writing. Gao et al [44] examined the differences between the writing generated by human and AI-generated text, such as ChatGPT, and the style of writing. The findings of their study revealed that the AI detector used accurately identified the abstracts that ChatGPT wrote. The researchers then checked for plagiarism which was found to be 0%.

## Methods

### Overview

This study aimed to examine the potential for, and concerns of, using ChatGPT to generate original manuscripts that would be accepted for publication by journals indexed in Web of Science and Scopus. We used ChatGPT to generate 4 versions of a full manuscript in the field of educational technology, specifically technostress and continuance intention regarding the use of a new technology. The faked research aimed to identify the relationship between the factors influencing teachers’ technostress and continuance intention to use a new technology continually. Moreover, using ChatGPT, we generated more than 50 abstracts for articles in the fields of social sciences and educational technology. These articles were previously published in journals (with an impact factor exceeding 2) indexed in Scopus Q1 and Q2. The researchers developed the prompts through long conversations with the model. For example, the first prompt was simple and asked for general information about the research topics. Then, the researchers developed the initial prompt based on the responses of the model by requesting more information, models, adding connector words, citations, etc.

### Description of the Full Articles

The generated article was composed of the main sections typically found in published research in journals indexed in Scopus (Q1 and Q2) and Web of Science (having an impact factor or Emerging Sources Citation Index). These sections included an Introduction (including the background to the study, the research problem, the purpose and contribution of the study, and research questions); a Literature Review (including the framework of the study); the Methodology (including the research design, tools, and data collection); Data Analysis (including suggested tables to be included in the study); and a Citation and References List. To improve the outcomes, we iterated the initial writing of these sections on 4 occasions. To obtain more detailed responses from ChatGPT, we began by providing simple prompts that gradually became more detailed. In all of the prompts, we asked ChatGPT to add citations within the text and to include a references list at the end of each section. These articles were named version 1, version 2, version 3, and version 4, respectively.

### Generated Abstracts

To generate the abstracts from published articles, we provided ChatGPT with a reference and asked it to generate an abstract from the article; this abstract was composed of no more than 200 words. In the prompt, we requested it to include the purpose of the study, the methodology, the participants, data analysis, the main findings, any limitations, future research, and contributions. We chose these terms based on the criteria for writing an abstract that would be suitable for publication in an academic journal. For the generated abstracts, we asked ChatGPT to provide us with abstracts from various fields of social sciences and educational technology. The criteria for writing such an abstract were that it should be suitable for publishing in an academic journal that is specified for the topic of the abstract and that it was composed of 200 words.

### Development of the Research Tool

We developed an evaluation form based on the review forms used in some journals indexed in Scopus and Web of Science. The purpose of the form was to guide the reviewers to review the full articles generated by ChatGPT. We submitted the form to potential reviewers who worked on behalf of some of the journals, to validate and ensure the content of the items in the form was good enough. We asked them to provide feedback by editing, adding, and writing their comments on the form. Some reviewers requested to add a new column to write notes, while others asked to separate the introduction into research ideas and research problems and to include the overall quality of the research questions. After developing the form, a pilot review was conducted with 5 reviewers regarding actual studies written by humans. The final version of the evaluation form is available in Multimedia Appendix 1.

### Focus Group Session

An online discussion group lasting 1 hour was conducted to discuss the quality of the abstracts and articles that were reviewed. An invitation was sent to 23 reviewers to attend the discussion session. Out of these 23 reviewers, 20 attended the online session. The discussion focused on how the reviewers judged the quality of the abstracts and articles, the content and sequence of ideas, and the writing style; 2 researchers moderated the discussion in the focus group session.

### Potential Reviewers

We intended to recruit 50 reviewers from different fields of social sciences and educational technology to review the abstracts and the 4 generated articles. We used the snowball technique to find potential reviewers to revise the abstracts and the 4 generated papers. We recruited 23 reviewers, all of whom held a PhD in different fields. They were from different countries and had published research in international journals. All of the reviewers had a similar level of experience in reviewing material for high-ranking journals.

All the reviewers were anonymous, and the review process followed a single-blind peer-review model. A total of 3 reviewers assessed each abstract individually, while 7 reviewers evaluated each of the 4 articles generated by ChatGPT using a blind peer-review process. The researchers requested that the reviewers give verbal feedback on the overall quality of the written articles, and all the reviewers submitted their reports to the first author.

In the beginning, the researchers did not inform the reviewers that ChatGPT had generated the content they would be reviewing. However, at the beginning of the focus group session, the moderator informed the researchers that the content they reviewed had been generated using the ChatGPT platform. All the reviewers were informed that their identities would remain anonymous.

### Data Analysis

The researchers analyzed the reviewers’ responses on the form using statistical analysis to identify the mean score of each item of the 4 versions of the research. They thereafter compared the results of the 4 articles to find the one-way ANOVA [45]. Moreover, the researchers analyzed the qualitative data comprising notes written in the note section on the evaluation form and the data obtained from the focus group session using thematic analysis [46]. The purpose of the qualitative data was to gain insights and a deeper understanding of the quality of the articles and abstracts from the reviewers’ perspective. The researchers used thematic analysis to analyze the qualitative data from the focus group session.

### Qualitative Data Analysis Procedures

The researchers recorded the focus group discussion session and one of the researchers took notes during the discussion. The audio file of the recorded session was transcribed. The researchers sent the text file to the participants to change, edit, or add new information. After 1 week, the researchers received the file without any changes. The unit analysis was a concept or idea related to the research questions; 2 researchers independently analyzed the qualitative data. After completing the analysis, the raters exchanged the data analysis files and found an interrater reliability of 89%. Any discrepancy between the coders, and the researchers, was resolved by negotiation to achieve agreement.

### Ethical Consideration

The researchers received approval to conduct this study from the Deanship of Scientific Research at the An Najah National University in Palestine (approval number ANNU-T010-2023). A consent form was obtained from participants in the focus group session and from the reviewers to use their records for academic research. Therefore, the statement was “Do you agree or not? If you agree please sign at the end of the form.” All the participants were informed that participation in the reviewing process and discussion in the focus group were voluntary and free without any compensation. Moreover, we informed them that their identity will be anonymous. At the end of the paragraph, the following sentence was added: “If you agree, we consider you signed the form, if not you can stop your participation in the study.”

## Results

### Reviewers’ Assessment of Research Quality and References in the 4 Study Versions

Based on the statistical data analysis performed by estimating the mean and SDs of the reviewers’ responses, as well as ANOVA [45], both Tables 1 and 2 represent the analysis results of the reviewers’ reports on the 4 study versions. All research stages were evaluated by the reviewers based on criteria developed by the researchers through the study of reviewing processes in high-impact journals such as Nature, Science, and Elsevier. The scale used to evaluate each stage of the research was as follows: 1=strongly disagree and 10=strongly agree. The midpoint of the scale represents the level at which a paper would be accepted for publication and was 5.5 in this study. Based on the findings presented in Tables 1 and 2, we found that the overall average quality of the research ranged from 5.13 to 7.08 for version 1 to version 4, respectively. Therefore, based on the midpoint criteria (5.5), not all of the papers would have been successful in being selected for publication. For example, version 2 scored less than the midpoint. Based on these findings, the weakest part of the developing studies, with less improvement in the 4 versions, was the cited references list. The value ranged from 4.74 to 5.61, leading to only 1 version (ie, version 4) of the generated studies being eligible for acceptance based on the references. However, upon checking whether the references listed were available, we found that only 8% of the references were available on Google Scholar (Google LLC/Alphabet Inc.)/Mendeley (Mendeley Ltd., Elsevier).

In addition, we found that the development of the prompts did not improve the quality of the research idea; for example, in version 3 (Table 1), the quality of the research idea was less than the quality of the idea in version 2. The major improvement could be seen in the writing of the literature review. We noticed an improvement between versions 1 and 4. The range was from 5.88 (version 1) to 7.32 (version 4).

According to data displayed in Table 2, there were differences in reviewing the 4 versions of the generated study (*P*=.02). The results differ because, according to the posttest (least significant difference), versions 3 and 4 exhibited greater significance (*P*<.001) compared with version 1. Therefore, there was no significant difference (*P*<.001) between versions 3 and 4, and both were superior to version 1. However, version 2 did not differ significantly (*P*<.001) from the other 3 versions. This result was due to the quality of the prompts used in the first version. The researchers used a simple prompt without any directions to write the abstract. Therefore, the reasonable difference between versions 3 and 4 was due to the difference in the stated prompts.

**Figure 1:**
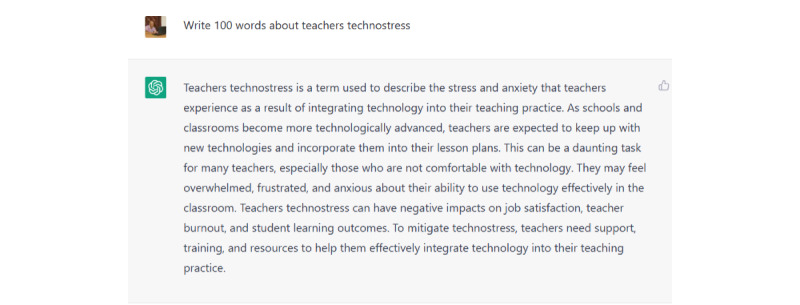
The simple question and ChatGPT's response.

**Figure 2:**
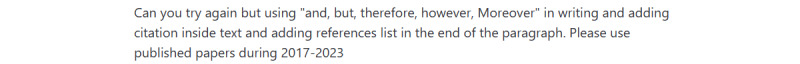
Developing the prompt to include connector words and add citation from specific years.

**Figure 3:**
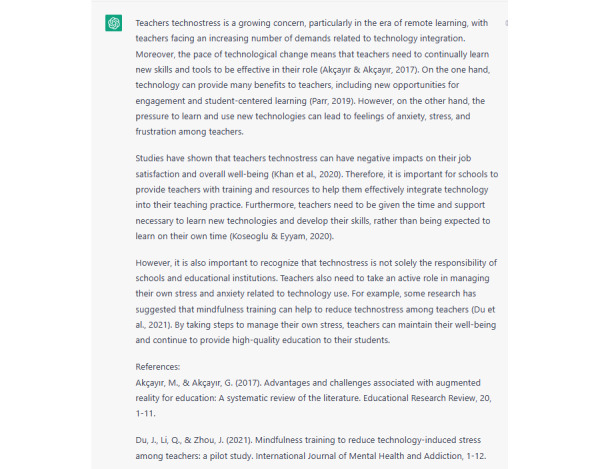
ChatGPT's response with more citations and references.

Moreover, there were significant differences (*P*<.001) in the research phases between the 4 versions, which were related to the development of the prompts used by the researchers to request responses from ChatGPT. An interesting finding in the citation and references list was that both versions 3 and 4 showed no differences in the development of in-text citations and the references list. Although the researchers used detailed prompts to train ChatGPT to improve in-text citations and references, there was no significant (*P*<.001) development. Hence, the response was simple—containing neither references nor connecting words as shown in Figure 1. We developed the prompt accordingly and managed to obtain a more professional response as illustrated in Figures 2 and 3. The whole idea is illustrated in Multimedia Appendix 2.

ChatGPT’s performance in version 4 is notable, not only in the overall quality but also in most research stages. However, in the focus group discussion, despite the improvement in the quality of research based on the enhancement of the prompts and the use of more context and detail, the reviewers mentioned that the quality of writing, especially the consequences and the use of conjunctions between ideas, was lacking and needed improvement. They mentioned that it was easy for the reviewers who had experience in reviewing articles to identify that the writing was accomplished using a machine rather than a human.

The reviewers in this study concluded that if journal reviewers have experience, they will realize that an AI tool, such as ChatGPT, has written the manuscript they are reviewing.

**Table 1:** The mean (SD) of the reviewers’ evaluation of the research stages of each version of the ChatGPT-generated research studies.

Descriptive and research			Mean (SD)
**Abstract**			
	Version 1	7.04 (0.71)	
	Version 2	7.26 (0.62)	
	Version 3	7.48 (0.51)	
	Version 4	7.57 (0.51)	
	Total	7.34 (0.62)	
**Research idea**			
	Version 1	6.30 (0.35)	
	Version 2	6.51 (0.40)	
	Version 3	6.47 (0.38)	
	Version 4	6.85 (0.33)	
	Total	6.53 (0.41)	
**Literature review**			
	Version 1	5.88 (0.36)	
	Version 2	6.43 (0.61)	
	Version 3	6.80 (0.57)	
	Version 4	7.32 (0.37)	
	Total	6.61 (0.71)	
**Methodology**			
	Version 1	5.59 (0.49)	
	Version 2	6.02 (0.51)	
	Version 3	6.61 (0.54)	
	Version 4	6.93 (0.53)	
	Total	6.29 (0.73)	
**Citation and references**			
	Version 1	4.74 (0.54)	
	Version 2	5.00 (0.50)	
	Version 3	5.41 (0.54)	
	Version 4	5.61 (0.69)	
	Total	5.19 (0.66)	
**Plagiarism**			
	Version 1	6.67 (0.36)	
	Version 2	6.78 (0.36)	
	Version 3	7.46 (0.30)	
	Version 4	7.83 (0.42)	
	Total	7.18 (0.60)	
**Total**			
	Version 1	6.01 (0.12)	
	Version 2	6.32 (0.28)	
	Version 3	6.61 (0.23)	
	Version 4	6.97 (0.23)	
	Total	6.48 (0.42)	
**Overall quality^a^**			
	Version 1	5.13	
	Version 2	5.8	
	Version 3	6.25	
	Version 4	7.08	

^a^Only means were compared.

**Table 2:** One-way ANOVA for the 4 versions of the study generated by AI.

Research components and source			Sum of squares			Mean of squares		*df*		*F*_3,88_		*P* value		Least significant difference	
**Abstract**									3.59		.02		Version 3 and version 4>version 1		
	BG^a^	3.77			1.26		3								
	WG^b^	30.78			0.35		88								
	Total	34.55			N/A^c^		91								
**Research idea**									9.06		<.001		Version 4>version 1, version 2, and version 3		
	BG	3.65			1.22		3								
	WG	11.80			0.13		88								
	Total	15.45			N/A		91								
**Literature review**									35.27		<.001		Version 1<version 2<version 3<version 4		
	BG	25.19			8.40		3								
	WG	20.95			0.24		88								
	Total	46.14			N/A		91								
**Methodology**									30.85		<.001		Version 1<version 2<version 3<version 4		
	BG	24.92			8.31		3								
	WG	23.70			0.27		88								
	Total	48.62			N/A		91								
**Citation and references**										10.90		<.001		Version 1<version 2<version 3=version 4	
	BG	10.68			3.56		3								
	WG	28.74			0.33		88								
	Total	39.42			N/A		91								
**Plagiarism**									53.36		<.001		(Version 1 and version 2)<version 3=version 4		
	BG	20.88			6.96		3								
	WG	11.48			0.13		88								
	Total	32.36			N/A		91								
**Total**									76.90		<.001		Version 1<version 2<version 3<version 4		
	BG	11.48			3.83		3								
	WG	4.38			0.05		88								
	Total	15.86			N/A		91								

^a^BG: between groups.

^b^WG: within the group.

^c^N/A: not applicable.

### Qualitative Findings

#### Findings of Best Practice When Using ChatGPT in Academic Writing

Based on the findings and an analysis of the reviewers’ reports from the focus group sessions, the optimal ways to use ChatGPT can be categorized into the following themes: Using Descriptive Prompts, Providing Context to the Prompts, Using Clear Language, and Checking The Outputs Obtained From ChatGPT.

#### Using Descriptive Prompts

During the development of the 4 articles, the researchers used a wide range of prompts, from simple to detailed, which significantly (*P*<.001) impacted the quality of each version of the research. For example, the researchers started the conversation with ChatGPT by using a simple prompt such as the one illustrated in Multimedia Appendix 2 (the prompts used in the study to generate the 4 versions). Using more detailed prompts improved the quality of the generated text (see data for version 4 in Table 1). By contrast, vague or simple prompts resulted in unrelated responses. For example, when we simply asked about the factors influencing technostress, ChatGPT responded that “TAM was the major framework used to understand technostress”; this is, however, untrue because technostress is related to technology acceptance and adoption.

#### Providing Context to the Prompts

It is important to provide context when requesting a platform (ChatGPT in this case) to summarize literature or generate a good research idea. Therefore, stating the context you are looking for will maximize your chances of receiving a strong, relevant response to your research. In the second version of the generated article, we noticed an improvement in the clarity of the research idea when we added the context to the prompt. In the first version of the article, we did not ask about teachers’ technostress, whereas in the second version, we solely focused on technostress. Adding context to the prompts was essential to provide us with an accurate response. Therefore, when researchers are experts in their field of study, technology such as ChatGPT can be a helpful tool for them. However, despite an improvement after adding context, the generated text continued to lack the quality and depth of academic writing.

Based on the experience of the researchers, their practices, and the responses of the reviewers in the focus group session, the quality of generating text using ChatGPT depended on the quality of the prompts used by the researchers. ChatGPT produced simple and basic content on a specific topic; in the context of this study, this was on the subject of teachers’ technostress and continuance intention to use a new technology. However, as practitioners, we must train ChatGPT to provide a high-quality and accurate response using detailed prompts. Here, we needed to train ChatGPT to provide us with such responses through the use of detailed prompts. For example, when we used a simple prompt to generate text about teachers’ technostress, the response was simple and without references or connecting words as illustrated in Figure 1.

The findings of the study presented in Tables 1 and 2, as well as the findings of the focus group session, show that the quality of the text generated using ChatGPT depends on the quality of the prompts used by the researchers. ChatGPT produced simple and basic content on a specific topic; in the context of this study this was about teachers’ technostress and continuance intention to use a new technology. The response was more developed with in-text citations, a references list, and the use of connectors between the sentences.

#### Using Clear Language

To obtain high-quality ideas for their research, researchers need to use simple, clear language containing comprehensive details about the subject matter of the research. We advise researchers to ensure that their prompts contain correct grammar and that they can make any corrections using ChatGPT before writing their prompts; this can be done by asking it to “correct the sentence or the paragraph.”

#### Checking the Outputs Obtained From ChatGPT

Humans are the experts and ChatGPT is a help tool. After receiving ChatGPT’s writing, it is therefore necessary to check it in terms of the quality of the content, the consequences of the ideas, in-text citations, and the list of references. On examination, we found that the in-text citations were incorrect and the references in the list were fabricated by the application; this therefore influenced the integrity of the research and brought us to the conclusion that ChatGPT can generate fake ideas and unauthentic references. Moreover, the predicted plagiarism in the generated text ranged from 5% to 15% depending on the general concepts used in the articles.

Furthermore, ChatGPT failed to cite in-text references, a common feature in academic writing. The references cited regarding all the queries were also grossly inappropriate or inaccurate. Citing inappropriate or inaccurate references can also be observed in biological intelligence, reflecting a lack of passion, and it is therefore not surprising to see a similar response from an AI tool like ChatGPT.

**Figure 4:**
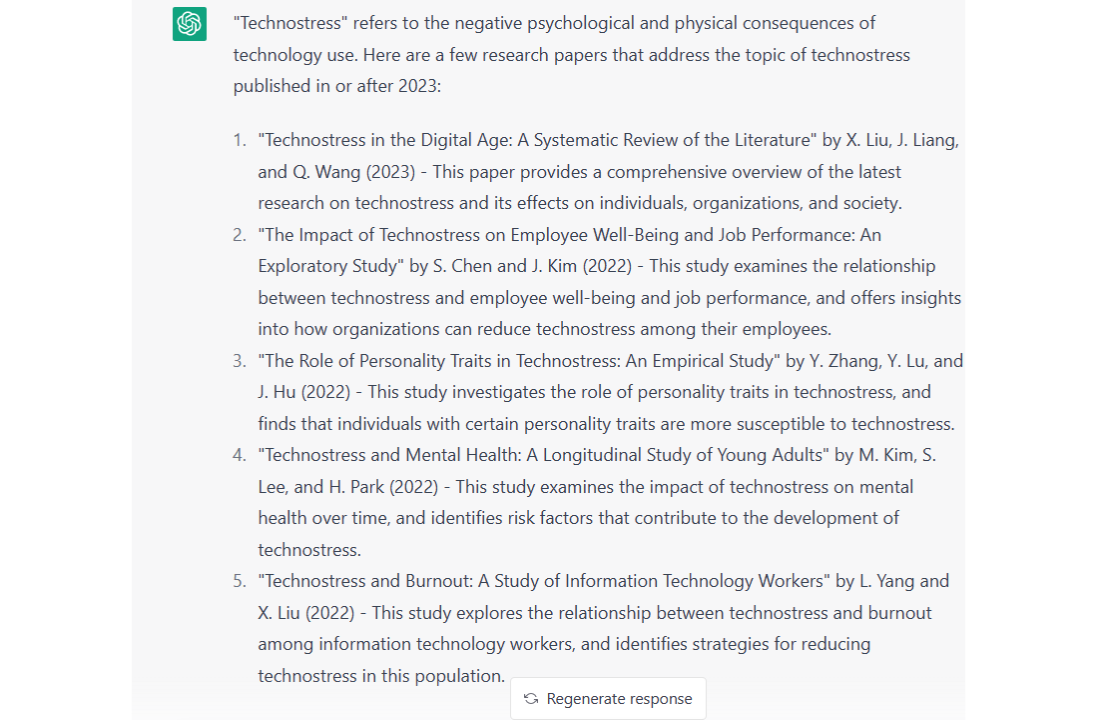
Misleading information about the framework.

In addition to the quality of the references cited, this number of references cited was a concern. This problem can often be seen in the context of academic writing from an audience that is not committed to the topic being researched. Another major flaw in the response from ChatGPT was the misleading information regarding the framework of the generated studies, as illustrated in Figure 4.

**Figure 5:**
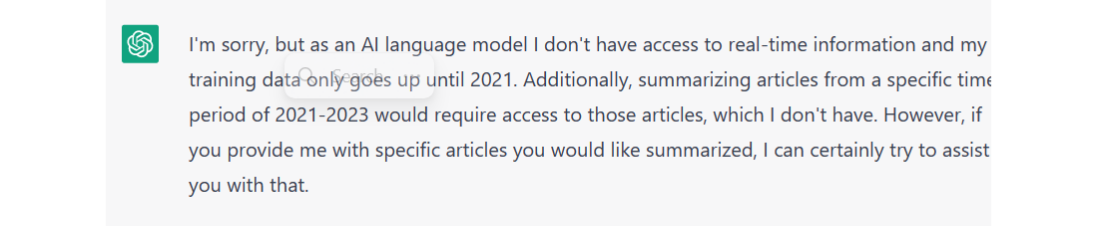
ChatGPT's response about its capability of accessing latest references.

We used Google Scholar to search for the titles of the cited articles but were unable to find them; this confirmed that ChatGPT fabricated the titles. Moreover, while asking ChatGPT to provide us with a summary of the findings of published articles from 2021 to 2023 in the field of technology integration in education, its response was “training only goes until 2021” as illustrated in Figure 5.

### Concerns About Using ChatGPT in Scientific Research

#### Overview

The potential reviewers in the focus group sessions raised various concerns in terms of using ChatGPT in scientific research. The researchers categorized these concerns into 4 themes, namely, Research Ethics, Research Integrity, Research Quality, and Trustworthiness of Citations and References.

#### Research Ethics

Most participants in the focus groups raised concerns about the use of ChatGPT due to the risk that it might lead to bias in the information provided in the responses. The reason for the biased responses is due to the use of words and concepts in the wrong context and, as reported by many participants, the application trying to convince researchers that it knows what it is doing. One of the reviews mentioned, “when I reviewed the fourth version, I noticed that all the information about using technology was a bright side and its effects were positive and bright when this was not the case. Based on my experience and research, technology also has a negative effect on users”.

Another ethical concern raised by the reviewers in the focus group discussion regarding the use of ChatGPT was false and misleading information while writing the literature review; this was reported by a majority of the reviewers. One reviewer confirmed that the inaccurate information provided by ChatGPT in scientific research could influence the integrity of the research itself.

Another issue relating to research ethics is authorship; this relates to the authors who have contributed to the research. The reviewers agreed that ChatGPT could not be regarded as a coauthor because it is not human and the generated responses it gives are from data that it has been trained to use. In addition, only a few reviewers connected research ethics to the whole process of conducting research; most viewed it as pertinent solely to the publication phase. They reported that ChatGPT could not be regarded as a corresponding author and could not work on the revision of the research, as can be seen from their reasons given below.

Copyright and ownership are additional ethical concerns when using ChatGPT in scientific research. This is because ChatGPT cannot sign an agreement to publish an article in a journal after it has been accepted for publication. This concern was identified by all the reviewers in the focus group discussion. One reviewer raised the question of ownership regarding the information and ideas generated by ChatGPT: Does the ownership lie with the researcher or with ChatGPT? At the end of the discussion, the reviewers and researchers involved in this study collectively determined that the responsibility for ensuring research accuracy and adherence to all research ethics primarily rested with human researchers. It was therefore the responsibility of researchers to ensure the integrity of their research so that it could be accepted and published in a scientific journal.

#### Research Integrity

Based on the findings of the reviewers’ reports, as well as the discussion among the researchers and the reviewers in the focus group, there was agreement among the participants that they could not have confidence in and trust ChatGPT when it came to scientific research. Some reviewers mentioned that the transparency of providing researchers with information is unknown; How does ChatGPT foster originality in ideas and idea-generation methods? Moreover, according to data in Tables 1 and 2, specifically with regard to citation and references list, ChatGPT fabricated references as well as providing inaccurate information about the theoretical framework of the developed studies, which was also noticed and reported by the reviewers.

#### Research Quality

All the reviewers confirmed that ChatGPT cannot generate original ideas; instead, it merely creates text based on the outlines it is trained to use. Moreover, some reviewers insisted that the information provided by ChatGPT is inconsistent and inaccurate. Therefore, it can mislead researchers, especially a novice who does not have much experience in their field. One reviewer mentioned that ChatGPT can generate reasonable information and provide a researcher with a series of ideas albeit without suitable citations or correct references.

The quality of abstracts generated from published articles was both poor and misleading. The quality was less than the midpoint for acceptance to be published in peer-reviewed journals. An example of the abstract is provided in Multimedia Appendix 3.

#### Trustworthiness of Citations and References

The majority of the reviewers reported that the research generated by ChatGPT lacked in-text citations, which can be considered a type of plagiarism. A few reviewers also expressed that ChatGPT fabricated the references listed. Some related examples are provided in Multimedia Appendices 4 and 5.

## Discussion

### Principal Findings

Using AI as an assistive tool in medical education validates its benefits in both medical education and clinical decision-making [47]. The findings of this study underscore the importance of training users to effectively utilize AI-generated text in various fields, particularly in alignment with recent studies advocating for the use of AI tools in medical education [48]. Therefore, AI-generated text tools can be integrated into the medical curriculum and can be used in medical research [49]. The findings of other studies have revealed that ChatGPT, as an example of an AI-generated text tool, can be used as a tool for performing data analysis and making data-driven recommendations/decisions, as demonstrated by the generation of the 4 articles [48,50]. However, the generated knowledge or decision needs approval from humans as mentioned previously [49,50]. AI-generated text assists researchers and medical educators in formulating their decisions by developing the prompts they use in their conversations with the AI tool.

One of the challenges associated with ChatGPT and other AI-generated text tools that emerged during this study is the potential for incorrect information and fake references and in-text citations, which could influence the quality of medical education negatively as reported by [51,52]. In addition, the credibility of scientific research deeply depends on the accuracy of references and resources; however, these are not currently available in AI tools.

### Conclusions

Based on the analysis of the reviewers’ reports, as well as the focus group discussions, we found that the quality of text generated by ChatGPT depends on the quality of the prompts provided by researchers. Using more detailed and descriptive prompts, as well as appropriate context, improves the quality of the generated text, albeit the quality of the writing and the use of conjunctions between ideas still need improvement. The study identified weaknesses in the list of references cited (with only 8% of the references available when searched for on Google Scholar/Mendeley). We also identified a lack of citations within the text. The study’s findings can inform the use of AI-generated text tools in various fields, including medical education, as an option to assist both practitioners in the field of medicine and researchers in making informed decisions.

In various countries, the issue of journal copyright laws and publishing policies regarding AI-generated text in scientific research requires an ethical code and guidelines; this is in place to address concerns regarding plagiarism, attribution, authorship, and copyright. In a scientific research collaboration with ChatGPT, the generated text should be iterated with human insight, allowing researchers to add their input and thus take ownership of the resulting work. This can lead to higher-level research studies using private data and systematic iteration of the research, making ChatGPT an e-research assistant when used appropriately. Previous studies on using AI-generated text have primarily focused on creating research abstracts and literature syntheses, while some have used AI in different aspects of conducting research. However, the functionality of AI text generators for scientific research highlights the need for the development of an ethical code and guidelines for the use of advanced technology in academic publishing, specifically in relation to concerns regarding plagiarism, attribution, authorship, and copyright. Although ChatGPT is highly efficient in generating answers, it draws information from various sources on the internet, raising concerns about the accuracy and originality of academic papers.

The practical implications of this study are the importance of using descriptive prompts with clear language, and the provision of a relevant context, to improve the accuracy and relevance of the generated text in different fields such as medical education. It is also important to check the outcomes obtained from ChatGPT and to be aware that AI-generated text may be recognized by experienced journal reviewers. The theoretical implications of the study highlight not only the potential of AI-generated text in academic writing but also the need for further research to address the limitations and challenges of this technology. Overall, this study provides insights for researchers and practitioners on how to effectively use ChatGPT in academic writing. Moreover, the tool can be used in the medical research field to analyze data; however, the researchers need to double-check the output to ensure accuracy and validity.

## Appendix

Multimedia Appendix 1 The final version of the evaluation form.

Multimedia Appendix 2 Example of the prompts used in the study.

Multimedia Appendix 3 Example of a generated abstract from a published article in a journal.

Multimedia Appendix 4 Example of the answer provided by ChatGPT about its source of information.

Multimedia Appendix 5 Example of the answer provided by ChatGPT about any possible intellectual property infringement.

## Abbreviations

AI: artificial intelligence

## Data Availability Statement

Data will be available upon request.
