# Supplementary figures and images for: The Potential and Concerns of Using AI in Scientific Research: ChatGPT Performance Evaluation
Source: JMIR Med Educ. 2023 Sep 14;9:e47049. doi: 10.2196/47049 (PMC10636627; doi:10.2196/47049)

**
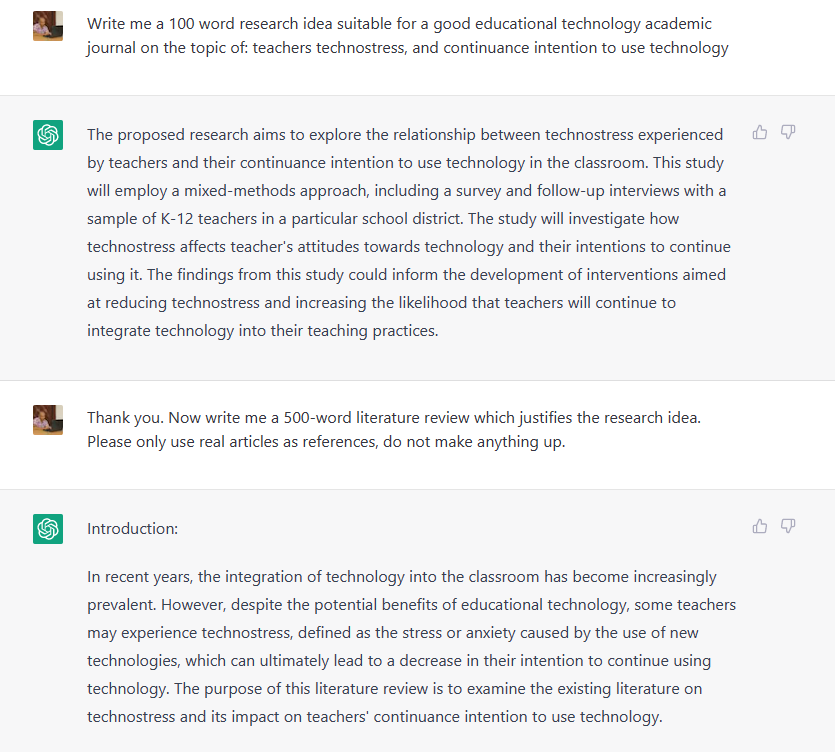
**

**
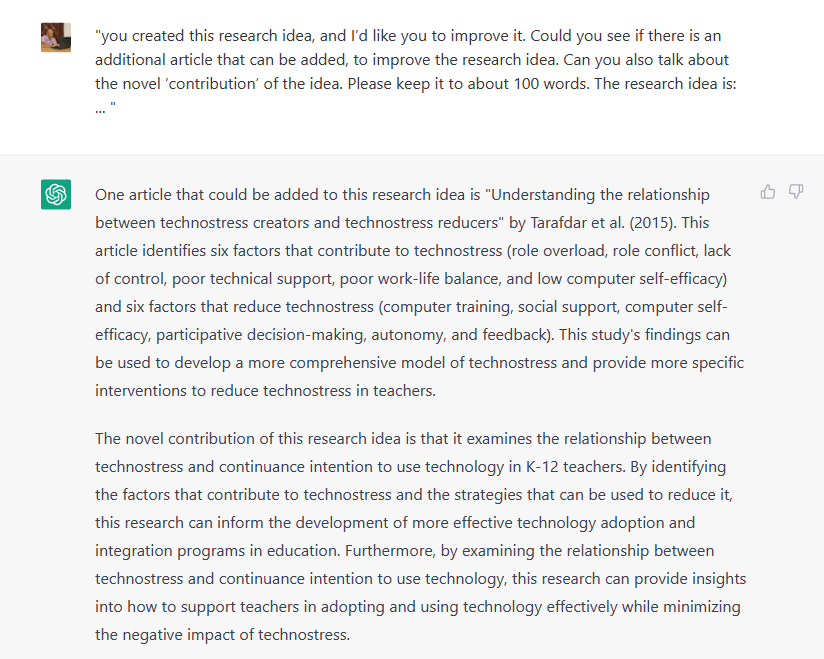
**

Supplement: Multimedia Appendix 2 [file mededu_v9i1e47049_app2.docx]

**
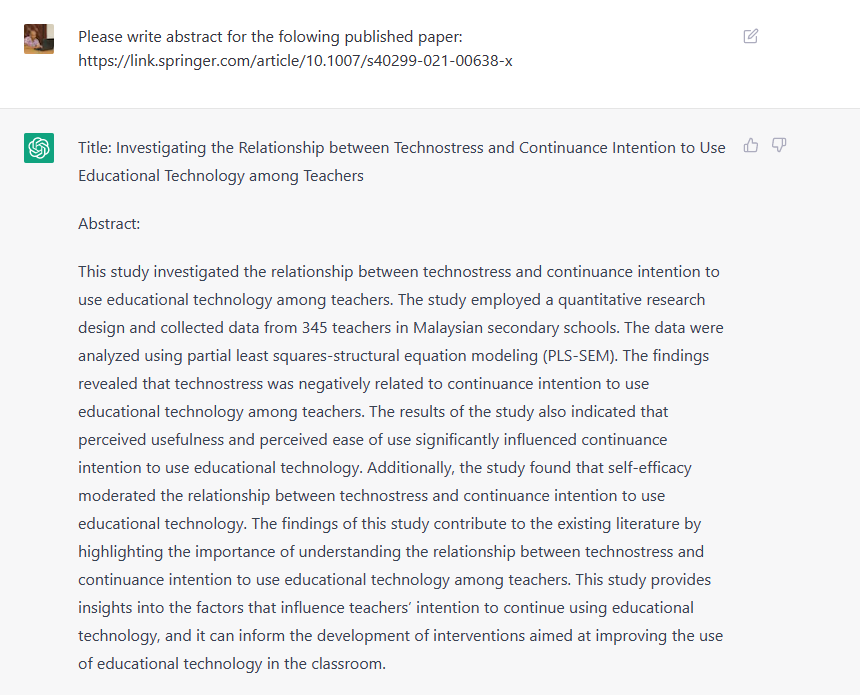
**

Supplement: Multimedia Appendix 3 [file mededu_v9i1e47049_app3.docx]

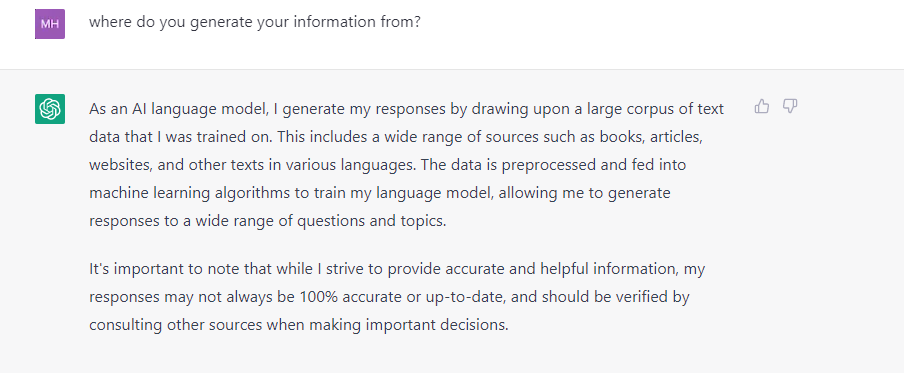

Supplement: Multimedia Appendix 4 [file mededu_v9i1e47049_app4.docx]

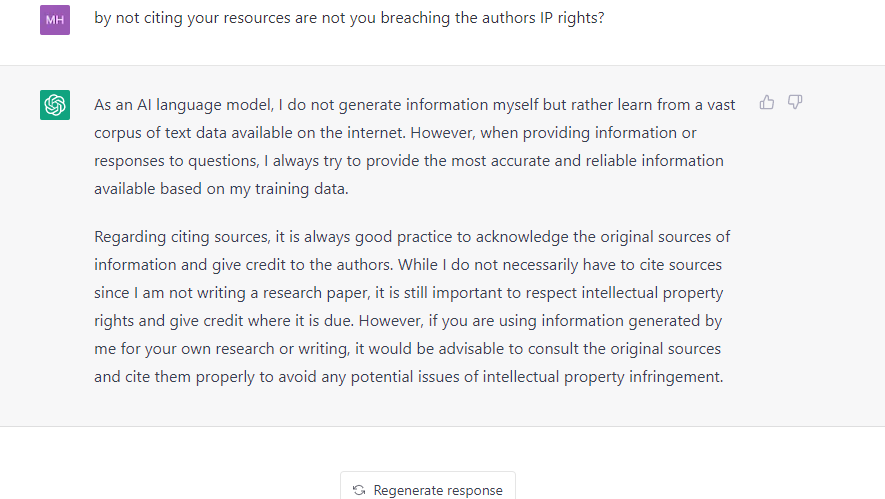

Supplement: Multimedia Appendix 5 [file mededu_v9i1e47049_app5.docx]
