# Supplementary material for: The Potential and Concerns of Using AI in Scientific Research: ChatGPT Performance Evaluation
Source: JMIR Med Educ. 2023 Sep 14;9:e47049. doi: 10.2196/47049 (PMC10636627; doi:10.2196/47049)
Supplement: Multimedia Appendix 1 [file mededu_v9i1e47049_app1.docx]

**Multimedia appendix 1**

**Dear Prof.**

The purpose of reviewing the abstracts and articles was to assess the quality of the scientific research. Therefore, we will use the data you provided us for scientific research. Your identity will be anonymous and all the data you will provide will be stored in an external drive protected with a password. Your participation in the study is free without any compensation. You are free to cancel your participation in the reviewing process at any time. Please if you agree to participate, sign below at the end of this form.

Thank you
